# Transcriptome Profiling of Leaves and Roots from Rooibos (*Aspalathus linearis*) Using Oxford Nanopore Sequencing

**DOI:** 10.3390/plants15111679

**Published:** 2026-05-29

**Authors:** Tanweer Beckett, Uljana Hesse

**Affiliations:** 1 Department of Biotechnology, University of the Western Cape, Robert Sobukwe Road, Bellville 7535, South Africa; 2 Institute for Microbial Biotechnology and Metagenomics, University of the Western Cape, Robert Sobukwe Road, Bellville 7535, South Africa

**Keywords:** RNA-seq, transcriptome, Oxford Nanopore, rooibos, *Aspalathus linearis*, long read, differential gene expression

## Abstract

Rooibos (*Aspalathus linearis*) is one of the few endemic South African plants that has achieved economic importance and international acclaim, mostly as a herbal tea. Plant production, limited to a small mountainous region in South Africa, is at risk as commercial rooibos longevity is in decline, mostly due to low stress tolerance. Transcriptome data can serve to identify molecular markers for improved stress response, which would speed up selection and facilitate the establishment of breeding programmes. Previously, rooibos leaf transcriptomes have been sequenced using Illumina, which yields short reads, hampering correct reassembly of full-length transcripts. Here, we established Oxford Nanopore-based, long-read transcriptome analysis for leaf and root samples from rooibos. We report on potential pitfalls in data pre-processing (PolyA tail trimming and rRNA removal), and compare two assemblers (RATTLE and RNA-Bloom2) and two clustering algorithms (VSEARCH and CD-HIT). The best assembly comprising 169,122 transcripts was generated using RNA-Bloom2 with short-read polishing, followed by CD-HIT clustering. Of the 95,054 predicted proteins, only 67% were also present in the Illumina dataset. The remainder comprised substantially shorter, mostly full-length sequences from a wide range of primary and secondary biosynthesis pathways. Functional annotation indicated that this transcriptome represents a high-quality, comprehensive resource for data mining. In the leaf fraction, comparative transcriptomics identified overexpressed rooibos transcripts potentially involved in photosynthesis, photorespiration and carbon fixation. In the roots, overexpressed transcripts encoded enzymes potentially involved in regulation of root growth and secondary metabolite biosynthesis. These transcripts may represent first targets for molecular marker development.

## 1. Introduction

Transcriptomics focuses on the analysis of sample-specific sets of RNA molecules expressed in an organism at a given time point under specific conditions. The data can be used for gene finding and to study gene expression. Comparison of transcriptomes from samples with contrasting phenotypes (e.g., when investigating varying compound production in different tissues of the same organism or in stress-resistant vs. stress-susceptible plants) permits identification of genes associated with the respective phenotypes. Comparative transcriptomics has been widely employed to identify molecular markers for desirable traits used for plant breeding (e.g., manifestation/regulation of morphological traits, developmental processes, abiotic stress and disease resistance, and desirable compound production). Marker Assisted Breeding is well established for most if not all major crops, including wheat, barley, corn, rice, soya, canola, cotton, diverse vegetables and fruits (reviewed in [1]). Recent technological advances in DNA/RNA/protein sequencing methods and big data analysis have also driven “omics” research (including genomics, transcriptomics, proteomics, and metabolomics) for neglected and underutilised “orphan” crops. In the context of Africa, these include diverse indigenous, nutrient-dense, climate-resilient grains (e.g., sorghum, finger millet), legumes (e.g., cowpea, lablab), as well as leafy and tuber vegetables (e.g., amaranth, sweet potato, cassava) to name but a few species (reviewed in [2,3,4,5,6,7,8].). Of particular interest are plant species that produce medicinally active compounds, where “omics” play increasing roles in elucidating the genes and metabolic pathways involved in compound production and pathway regulation, but also in quality control, safety evaluation, and authentication [9,10].

Rooibos (*Aspalathus linearis*) is one of the few endemic South African medicinal plant species that has reached economic importance and international acclaim, primarily as a herbal tea [11]. Production volumes reached over 22,000 t in recent years, nearly half of which is exported to over 50 countries throughout the world (including Japan, France, Germany, the Netherlands and the UK as top importers). This leguminous plant is native to the Cape Floristic Region of South Africa. It is found throughout the Cederberg mountains and on the Suid Bokkeveld plateau (Western and Northern Cape provinces), where it grows in nutrient-poor, acidic soils [12]. Due to its variability in morphology and reproductive strategies, *A. linearis* is regarded as a species complex. It is primarily divided into sprouters—fire-resistant growth types that resprout from lignotubers and include the Southern, Northern, Grey, and Nieuwoudtville Sprouters—and seeders, fire-sensitive growth types that rely on seed propagation and include the Red, Tree, Wupperthal, and Black Types [13,14,15]. Rooibos is known for its rich content of phenolic compounds, some of which are species-specific. Aspalathin, a dihydrochalcone so far only reported in *A. linearis* and its sister species *A. pendula*, is a potent antioxidant with anti-inflammatory, antidiabetic, and cardioprotective properties [16,17,18]. PPAG (Z-2-(β-D-glucopynosyloxy)-3-phenylpropenoic acid), so far only reported in rooibos, shows antidiabetic and cytoprotective activities, and can enhance glucose uptake, improve glucose tolerance, and protect pancreatic beta cells and cardiac tissues [19,20,21].

For large-scale commercial production, rooibos seeds are sown in autumn (between February and March) into seed beds in dedicated nurseries. After four to six months (June–August), the seedlings have developed into 10–30 cm tall plants with one to four supple stems, a well-structured root system (including a thin, long tap root that can reach over 1 m into the ground, and cluster roots that are most prolific in the top (0–30 cm) soil layer), as well as ample young leaves that already produce major phenolic compounds [22]. At this stage, the seedlings are transplanted from the nurseries to the commercial fields: blocks of 30–50 seedlings are dug up from the seed bed (soil depth ≈ 30 cm) and transported to the field where the strongest ones are planted individually into rows (8000–10,000 plants/ha). At one year of age, the bushes are cropped to promote branching. The first harvest for commercial use is typically conducted in February–March of the second growth year. On average, the rooibos plants can be harvested annually for another three to five years, depending on plant survival.

However, rooibos production is threatened by changes in seasonal precipitation and temperatures associated with climate change [12,23]. The commercial rooibos plants used for large-scale cultivation today were originally derived from a comparatively small pool of wild Red Type plants [15], and the minuscule seeds used for propagation are often collected from plants that produce high amounts of them (a stress avoidance mechanism). Current efforts therefore focus on improving the gene pool for commercial rooibos production towards improved stress resistance. Knowledge on the genes associated with these traits is a prerequisite for the development of molecular markers.

Recent efforts have been focusing on generating genome and transcriptome data resources for rooibos [24,25,26]. The rooibos genome size has been estimated to range between 1.03 Gbp and 1.24 Gbp, based on k-mer and flow cytometer analyses, respectively [24]. For the sequencing of the genome, two technologies were implemented: Illumina and Oxford Nanopore Technology (ONT) [25]. Illumina is a high-throughput sequencing platform that yields copious amounts of high-quality but very short (50–300 b) reads from fragments of DNA molecules. These short reads often fail to span long repetitive regions, rendering reassembly of large, heterozygous genomes with a high proportion of repeats, such as the rooibos genome, challenging. The ONT platform produces large numbers of long reads (>100 kb in length), but the quality is substantially lower compared to Illumina. For the rooibos genome, the assembly of long ONT reads and subsequent polishing with Illumina reads was found to substantially improve assembly completeness, contiguity and accuracy [25]. The previously published rooibos transcriptomes had been sequenced using only the Illumina platform [26]. Moreover, the samples were only generated from leaves. Since many secondary metabolites are produced in the roots, the corresponding genes may not be represented in these datasets.

This study aimed to (1) establish ONT-based long-read sequencing for rooibos transcriptome analysis, (2) construct a comprehensive rooibos reference transcriptome that permits mining for genes expressed in leaves and roots, and (3) explore whether the generated ONT data is suitable for comparative transcriptome analyses and can be used to shortlist rooibos transcripts potentially upregulated in leaves and roots for future laboratorial investigations.

## 2. Materials and Methods

### 2.1. Plant Material

Leaf and root samples were obtained from a single, robust, healthy, six-month-old rooibos seedling dug up from the nursery field at BBV Boerdery Ltd. (Citrusdal, South Africa) in August 2022. The single 26 cm long stem had many side branches and green leaves, and the root system (harvest depth 30 cm) was well developed. The seedling therefore represented a suitable representative typically used for transplanting. The leaves and roots were flash-frozen on site using liquid nitrogen, transported to the laboratory in a vapour dewar filled with liquid nitrogen, and stored at −80 °C.

### 2.2. Total RNA Extraction

For total RNA extraction, the leaves and roots were ground separately in liquid nitrogen using flash-frozen mortars and pestles. Thereafter, 250 mg of the powder was added to a 2 mL Eppendorf tube and overlayed with 1.5 mL of pre-heated RNA extraction buffer (100 mM Tris-HCl, pH 8.0; 25 mM EDTA; 2 M NaCl; 2% (*w*/*v*) PVP; 2% (*w*/*v*) cetyltrimethylammonium bromide; and 6% (*v*/*v*) β-mercaptoethanol, added to the extraction buffer just before use). The tubes were vortexed, incubated at 65 °C for 30 min with intermittent mixing, and then centrifuged at 16,000× *g* for 10 min. The supernatant was transferred to a phase-lock gel tube (5Prime) (Whitehead Scientific, Cape Town, South Africa), an equal volume of chloroform was mixed in by inversion, and phase separation was completed by centrifugation at 16,000× *g* for 10 min at room temperature. The aqueous phase was transferred to a new phase-lock gel tube and the organic purification was repeated. Thereafter, the aqueous phase was transferred to a fresh 2 mL Eppendorf tube, LiCl was added to a final concentration of 2 M, and the sample was left to precipitate at 4 °C overnight. The following day, the RNA was pelleted by centrifugation at 16,000× *g* for 10 min at 4 °C. The pellet was washed with 75% ethanol to dissolve any remaining salt, followed by 100% ethanol to fasten water evaporation. The RNA was then eluted in 50 µL nuclease-free water. RNA quality and quantity was determined using a NanoDrop spectrophotometer (Thermo Fisher Scientific, Waltham, MA, USA), and by electrophoresis on a 1% agarose gel in 1× TBE.

Each sample was treated with DNAse using the DNA-free™ Kit from Ambion (Life Technologies, Carlsbad, CA, USA) using 3 µg of total RNA and following manufacturer’s instructions. To assess the success of DNA removal, RNA samples were screened by amplifying the internal transcribed region (ITS) using PCR. Each 25 µL PCR reaction contained 100 ng of RNA, 5 µL 5× OneTaq^®^ Buffer, 0.5 µL 10 mM dNTP Solution Mix (New England BioLabs, Ipswich, MA, USA), 0.125 µL OneTaq^®^ DNA Polymerase (5000 units/mL, New England BioLabs), 0.5 µL 10 mM ITS_17SE primer (Inqaba Biotech, Pretoria, South Africa), 0.5 µL 10 mM ITS_26SE primer (Inqaba Biotech), 1.25 µL DMSO, and nuclease-free water. PCR was performed using an initial denaturation cycle at 94 °C for 3 min; 34 cycles of denaturation at 94 °C for 30 s, annealing at 55 °C for 30 s, and extension at 72 °C for 1.5 min; and a final extension cycle at 72 °C for 7 min. The PCR reactions were assessed on a 1% agarose gel prepared with 1× TBE.

### 2.3. cDNA Sequencing

In total, four cDNA libraries were prepared using two kits from Oxford Nanopore Technologies (ONT, Oxford, UK): SQK-PCS109 and SQK-PCS111. The libraries were generated, strictly following manufacturer’s instructions using 50 ng of total RNA for the SQK-PCS109, and 200 ng of total RNA for the SQK-PCS111 kits. For PCR, 18 cycles with an extension time of 12 min were performed. After PCR and just before the addition of 1 µL of exonuclease I, 0.25 µL from each of the four PCR tubes per sample were pooled to make up 1 µL. This aliquot was analysed on a 1% agarose gel to inspect the amount and integrity of the cDNA. The cDNA quantity was determined using the Qubit^®^ dsDNA HS assay kit (Thermo Fisher Scientific). The four adapter-ligated cDNA libraries were loaded onto four FLO-MIN106 R9.4.1 spotON flow cells (ONT) following the ONT’s priming and loading protocol, and were sequenced using an MK1C device (ONT). The sequencing runs were conducted for a maximum of 72 h or until all pores were irreversibly used up.

### 2.4. Data Processing

The computational analyses were completed using either the computer cluster at the Centre for High Performance Computing (CHPC, Cape Town, South Africa) or the European Galaxy Server (usegalaxy.eu).

The fast5 data files from the four sequencing runs were basecalled individually in high-accuracy mode using Guppy v6.1.7 (https://nanoporetech.com/software/other/guppy/history?version=6-1-7, accessed on 4 May 2026) with the dna_r9.4.1_450 bps_hac.cfg configuration file and a per-read-average Q-score cut-off of 7. All reverse reads were reverse complemented using Pychopper v2 (https://github.com/epi2me-labs/Pychopper, accessed on 4 May 2026) with default parameters, specifying the respective library construction kit (-k PCS109/PCS111). In the process, Pychopper also trimmed 5′ and 3′ reverse-transcription primers, PCR primers and sequencing adapters. Cutadapt v3.4 [27] was used twice with default settings to trim residual 3′ polyA sequences and GT or AC dimer repeats that were embedded into polyA tails. Reads < 200 b were filtered out using NanoFilt [28] by specifying the minimum read length -l = 200.

To filter the datasets for rRNA reads, a protocol involving SortMeRNA v3.4.7 [29] with an e-value threshold of 1 × 10^−10^, Minimap2 [30] using the parameters -ax map-ont, and the SILVA ARB Magnoliophyta database [31] was established. Initially, only SortMeRNA was employed to identify rRNAs in the four datasets using default parameters and the SILVA ARB database of large and small rRNA subunits [31]. However, we discovered that many filtered reads were actually valid mRNAs. Mapping the reads to the same database using Minimap2 and setting parameters to -ax map-ont confirmed excessive loss of mRNA sequences. Changing the database (SILVA ARB Magnoliophyta) and increasing e-value threshold for SortMeRNA to 1 × 10^−10^ resulted in comparable results between the two filtering approaches and undetectable loss of mRNA sequences (as verified manually on 100 sequences per dataset). Ultimately, the rRNA-depleted datasets were generated by removing reads that were aligned to the Magnoliophyta database by both tools, resulting in four library-specific datasets with rRNA reads (rRNA+) and four datasets without (rRNA−). For efficient transcriptome assembly, all reads shorter than 300 b were removed from the datasets using NanoFilt.

### 2.5. Transcript Assembly

For assembly, the four library-specific datasets were combined into two large rRNA+ and rRNA− datasets, respectively, and assembled using RATTLE [32] and RNA-Bloom2 [33]. By default, RATTLE clusters reads into gene clusters without splitting the isoforms of each respective gene, and considers that reads are not necessarily in forward direction. In this study, RATTLE’s “cluster” command was run with the -iso flag to separate the isoforms, and the -rna flag to disable reorientation of the reads. The second RATTLE command “correct” was run using the arguments specifying the output file of the “cluster” algorithm (cluster.out) and the original MinION reads file for error correction (in fastq format). The final RATTLE command “polish” was set to input the output file from the “correct” step (consensi.fq) and the -rna flag. RNA-Bloom2 was first run with the -long flag (for ONT data) and the -stranded flag (indicating that all reads are in the forward 5′- 3′ orientation). Another RNA-Bloom2 assembly was run using the same parameters and polishing with Illumina reads generated from a commercial rooibos plant in a previous study [26].

To reduce redundancy, the four RNA-Bloom2 assemblies were subjected to cluster analyses using either the clustering module of VSEARCH, Galaxy version 2.8.3.0 [34], with parameters -cluster-fast -usersort no -iddef int -id 0.9 -cons_truncate no -qmask dust; or CD-HIT, Galaxy version 4.8.1 [35], with parameters cd-hit-est -c 0.9 -n 10. The quality of the 14 resulting assemblies was assessed using rnaQUAST [36] and BUSCO with the Fabales lineage database [37]. Based on the assembly statistics and number of BUSCOs, the RNA-Bloom2 rRNA-depleted polished assembly that was clustered using CD-HIT (hereafter referred to as RB2 transcriptome) was used for annotation and further analyses.

### 2.6. Protein Prediction and Annotation

TransDecoder [38] was used to identify the longest open reading frames (ORFs), setting a minimum protein length (-m) of 100 amino acids. Protein prediction was conducted on ORFs that showed homology to sequences in the Pfam-A and UniProtKB databases. For functional annotation the predicted proteins were analysed using EggNOG-Mapper V2.1.13 [39] with default settings. The following annotations were computed for each protein sequence: (1) ortholog mapping-based KO assignment using Diamond and the EggNOG database v5.0.2; (2) protein family annotations using Pfam; (3) K-number, EC number and biosynthetic pathway annotations using KEGG Mapper; and (4) carbohydrate active enzyme identification using CAZy. KEGG Mapper uses K-numbers that are linked to the KOs (universal ortholog groups in KEGG) to extract associated pathway module information. This mapping algorithm does not discriminate between the pathways in organisms from different kingdoms of life. To obtain an overview on the annotation completeness of plant-specific pathways, a custom KEGG pathway module database was generated as follows: (1) the KEGG module pathways of diverse plant species from the Magnoliopsida were individually investigated on KEGG-BRITE (https://www.kegg.jp/brite/br08611, accessed on 4 May 2026) to identify species with the most complete and/or complementing pathway annotations; (2) the list of pathway modules (including the M-numbers and names) for *Glycine max*, *Phaseolus vulgaris*, *Cicer arietinum*, *Theobroma cacao*, *Arabidopsis thaliana*, and *Oryza sativa* were copied from the species-specific pathway modules page; (3) the resulting dataset was organised into parental pathway modules (i.e., carbohydrate metabolism, energy metabolism, etc.); and (4) the submodules within each parental pathway module were deduplicated using a custom awk script. The resulting plant-specific KEGG pathway module database contained a total of 258 pathway modules. KEGG pathway module completeness for the RB2 transcriptome was determined by providing the transcripts and their respective K-numbers to the reconstruction tool embedded in KEGG Mapper. The result was an interactive HTML, which reported the presence or absence of required enzymatic blocks for each pathway module. Only KEGG modules present in the deduplicated plant module database were considered. A customised awk script was used to classify the modules as ‘complete’, if all required blocks were present, or ‘incomplete’ if one or more blocks were missing.

### 2.7. Orthology Analyses

OrthoFinder v2.5.5 [40] was used with default parameters to identify shared orthologous groups (OGs) between the RB2 transcriptome and the Illumina-based rooibos transcriptome A generated from a commercial rooibos plant [26]. For this, the Illumina transcriptome was first re-analysed using TransDecoder to update the encoded protein dataset. Subsequently, the proteomes for various model legume species, namely *Glycine max* (v2.1; GM), *Lupinus angustifolius* (Tanjil v1.0; LA), and *Medicago truncatula* (MtrunA17r5.0_ANR; MT), as well as the non-legume *Arabidopsis thaliana* (TAIR 10.1; AT), downloaded from RefSEQ or ENSEMBL, were included in the analysis. To illustrate the results, R was used to generate Venn diagrams and UpSet plots.

### 2.8. Differential Expression Analyses

Differential expression analysis was conducted using the R packages ‘EdgeR’ [41] and ‘DESeq2′ following the program manual instructions [42]. The samples L109 and L111 served as technical repeats for the group ‘leaves’ and the samples R109 and R111 were used as technical repeats for the group ‘roots’. Because the main aim of this study was the establishment of ONT sequencing and data analysis protocols (specifically, comparison of two cDNA sequencing kits available at the time), technological replicates were given priority and no biological replicates were generated.

First, the reads from the four cDNA libraries (L109, R109, L111, R111) were mapped to the RB2 transcriptome assembly using Minimap2 v2.30 with the parameters: -ax map-ont -p 0 -N 10. The four bam files produced by Minimap2 were used as input for NanoCount [43] to quantify the number of reads that mapped per transcript and generate a count matrix. To ensure a fair comparison between EdgeR and DESeq2, the count matrix was filtered so that only rows where the sum of the counts across the four samples was ≥10 were retained. The internal filtering parameters for EdgeR and DESeq2 were therefore disabled. For EdgeR, library sizes were normalised using the default trimmed mean of M-values (TMM) method, which corrects for compositional differences between samples, assuming that the majority of genes are not differentially expressed. Dispersion was estimated using the ‘estimateDisp’ function with ‘robust=TRUE’, differential expression was then tested using the quasi-likelihood F-test with glmQLFit and glmQLFTest with the parameter robust=TRUE. For DESeq2, libraries were normalised using the median-of-ratios method with parametric dispersion fitting, and differential expression was tested using the Wald test. Beta priors were disabled. For both tools, transcripts were considered significantly expressed at a false discovery rate (FDR) of <0.05, calculated using the Benjamini–Hochberg procedure to control for multiple testing. The transcripts with a log2 fold-change (logFC) ≥ 1 were classified as overexpressed in leaves, and those with a logFC ≤ −1 were classified as overexpressed in roots.

### 2.9. Over-Representation Analyses

Over-representation analyses for gene ontology (GO) terms and Kyoto Encyclopedia of Genes and Genomes (KEGG) pathways were completed for overexpressed leaf and root transcripts using the R package ‘clusterProfiler’ [44], specifically the ‘enricher’ and ‘enrichKEGG’ functions, respectively. For GO term enrichment analysis, the EggNOG Mapper annotation file was used to produce a ‘term-to-gene’ table, where the first column contained the GO terms and the second column the corresponding IDs of the differentially expressed leaf and root transcripts. Then, the GO.obo file from the obolibrary (https://purl.obolibrary.org/obo/go.obo, accessed on 4 May 2026) was used to create a ‘term-to-name’ table, where the first column contained the GO terms and the second column their corresponding descriptions stored under “is_a” in the GO.obo file. Thereafter, the leaf and root DEG datasets were analysed using the package’s ‘enricher’ function with *p*-value and q-value cut-offs set to 0.05. Subsequently, GOMCL [45] was used to cluster redundant GO terms and to generate cluster networks. For KEGG pathway enrichment analyses, the K-number annotations for the differentially expressed leaf and root transcripts were extracted from the EggNOG annotation file and analysed using the ‘enrichKEGG’ function with *p*-value and q-value cut-offs set to 0.05. Only plant-specific KEGG module pathways were investigated.

Enrichment of protein families and functional domains was analysed by determining the frequencies of Pfam IDs for the differentially expressed leaf and root transcripts, respectively.

## 3. Results

### 3.1. RNA Extraction, Library Construction and Sequencing

**Figure 1:**
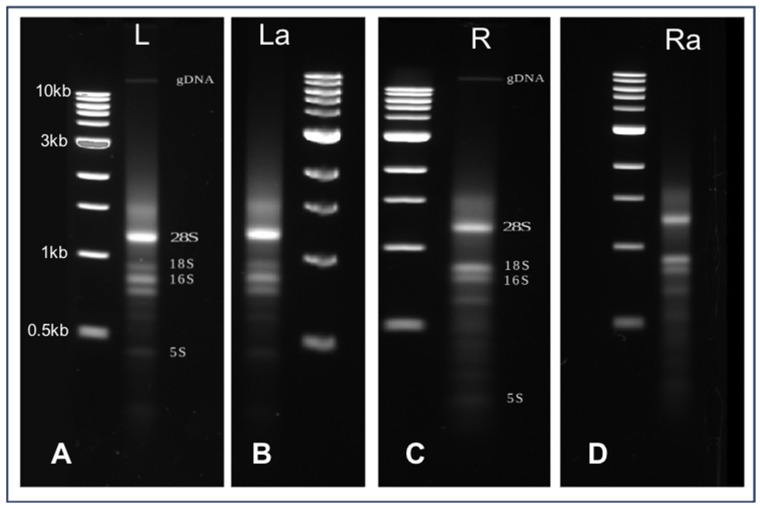
RNA gels for the leaf and root samples from the rooibos seedling ((**A**): leaves before, (**B**): leaves after, (**C**): roots before, (**D**): roots after DNAse treatment).

The first objective was to generate high-quality RNA for MinION sequencing. Using the same amount of starting material (250 mg), the seedling leaf sample yielded nearly three times more total RNA than the root sample (L: 60 mg at 1200 ng/µL; R: 20 mg at 393.7 ng/µL). The RNA quality was excellent: the absorbance readings for both samples were within the thresholds for pure RNA, although the A260/A280 ratio (L: 2.22; R: 2.08) was slightly lower, and the A260/A230 ratio (L: 2.23; R: 2.40) was slightly higher in the root sample as compared to the leaf sample. DNA that co-precipitated with the RNA (≈1.6 mg at 33 ng/µL in both leaf and root samples) was successfully removed, as verified by PCR and gel electrophoresis (Figure 1).

RNA sequencing of leaves and root samples was conducted using two cDNA library preparation kits from Oxford Nanopore Technologies: PCS-SQK109 (samples L109 and R109) and PCS-SQK111 (samples L111 and R111). In total, the four runs produced 53.6 Mio reads (min QS 7) amounting to 51.6 Gb of data (Table 1), ranging between 12 Mio and 20 Mio reads per flow cell (not counting R109, which was damaged during priming). The reads produced with the PCS-SQK109 kit were notably shorter than the reads generated with the PCS-SQK111 kit, as indicated by the lower median read lengths. Subsequently, only 42% of the reads from the PCS-SQK109 libraries were retained after quality processing: 23% of the reads were lost after Pychopper and another 35% after NanoFilt analysis. In contrast, 84% of the reads from the PCS-SQK111 libraries passed these filtering steps. Here, only 6% of the reads were removed by Pychopper and another 10% by NanoFilt. Ultimately, 37.6 Mio quality-filtered reads were used for rRNA filtering.

Filtering for rRNA reads was not straightforward. Manual analyses showed that SortMeRNA tended to remove substantial numbers of non-rRNA reads: when using the entire SILVA database and default parameters, only 28.6 Mio reads (76%) were retained (Table 1). When using the program with the Magnoliophyta database and an e-value threshold of 1 × 10^−10^, 34.2 Mio reads (91%) were recovered. Verification using Minimap2 indicated similar results. Subsequently, the best approach for filtering rRNA reads was to remove only those reads that mapped to the Magnoliophyta subset of the SILVA database using SortMeRNA with an e-value threshold of 1 × 10^−10^ and Minimap2. Using this approach, 31.2 Mio reads (83%) were retained.

### 3.2. Transcriptome Assembly

For transcriptome assembly, two assembly programs (RATTLE and RNA-Bloom2) were investigated. RATTLE consistently exceeded the allotted wall time (48 h per step) when provided with datasets larger than 32 Mio reads. Therefore, the four datasets (L109, L111, R109, R111) were filtered once more to remove all reads shorter than 300 b, yielding two final datasets: (1) rRNA+ with 31 Mio reads, and (2) rRNA− with 28 Mio reads. In addition, the effect of polishing the long reads with short Illumina reads before assembly (offered by RNA-Bloom2), and clustering the RNA-Bloom2 assembly using either VSEARCH or CD-HIT, was investigated. In total, 14 transcriptomes were generated (Table 2).

When used with default parameters, RATTLE assembled 57,270 transcripts (rRNA+) and 58,497 transcripts (rRNA−). The mean, median and the longest transcript lengths were not substantially different between the two assemblies (averaging 1844 b, 1639 b, and 10,129 b, respectively). The BUSCO statistics were also similar: on average, 78.6% of the BUSCO hits were complete (16% of which were duplicates), and 16% of the BUSCO hits were missing.

RNA-Bloom2 produced nearly four times as many transcripts as RATTLE (≈227,000 transcripts with the rRNA+ dataset, and ≈212,000 transcripts with the rRNA− datasets). The mean and median transcript lengths were notably shorter (1430 b and 1254 b, respectively), but BUSCO scores were substantially higher (87.7% to 90.1% of the BUSCOs were complete). Removal of rRNA reads reduced the maximum transcript length (on average from 13,198 b to 11,343 b). Polishing did not affect the transcript numbers or length statistics, but improved BUSCO scores, yielding ≈ 2% more complete BUSCOs.

**Figure 2:**
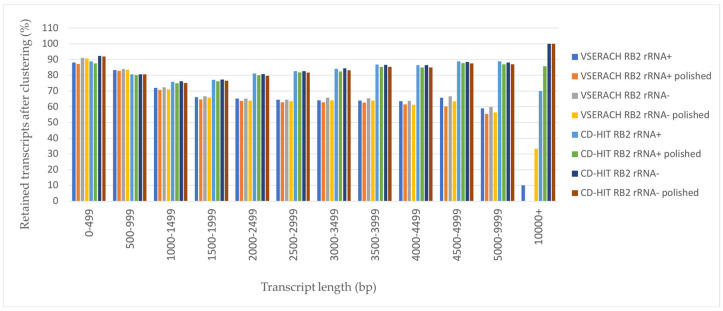
Proportion of transcripts retained after clustering using VSEARCH or CD-HIT of the RNA-Bloom2 (RB2) assemblies (either with or without rRNA (rRNA+/rRNA−) sequences, unpolished or polished with Illumina data).

Because the RNA-Bloom2 transcriptomes were likely assembled incompletely (as indicated by the high number of transcripts and high proportions of duplicated BUSCOs), the datasets were clustered using either VSEARCH or CD-HIT, yielding assemblies with 162,087 ± 5533 and 175,522 ± 6213 contigs, respectively. In comparison to VSEARCH, CD-HIT produced somewhat longer transcripts of better quality, as indicated by higher proportions of complete BUSCOs. Again, rRNA filtering reduced the maximum transcript lengths, while polishing improved BUSCO statistics. Analysis of read length distributions showed that for shorter transcripts (up to 1 kb), the proportion of retained transcripts was similar between the two programs (80–92%). For longer transcripts, however, CD-HIT consistently retained a higher proportion of transcripts than VSEARCH (Figure 2).

Despite reducing the input dataset size, RATTLE required substantial computer resources, each of the three assembly steps taking ≈48 h to complete. In comparison, RNA-Bloom2 finished all assemblies within a maximum of 9.5 h. Clustering with VSEARCH was fast, requiring less than a minute of computing time. CD-HIT, on the other hand, required at least 7 days to complete the analysis. Filtering the datasets for rRNA reads substantially reduced running times.

Since rRNA transcripts may interfere with differential expression analyses, the rRNA-depleted, polished RNA-Bloom2 assembly clustered with CD-HIT (CD-HIT RB2 rRNA− polished, hereafter referred to as RB2) was used for downstream analysis.

### 3.3. Transcriptome Annotation

Of the 169,122 transcripts, 95,054 (56%) were predicted to encode proteins. For functional annotation, the transcripts and the predicted proteins were compared to functionally characterised protein sequences in the SwissProt and KEGG databases using KAAS and EggNOG-Mapper (Table 3). In total, only 10% of the transcripts could be annotated using KAAS. In contrast, the majority of the predicted protein sequences (92%) showed significant similarities to protein sequences in the Swissprot database, and 94% were annotated by EggNOG-Mapper with K-numbers, GO terms and/or Pfam annotations. It is worth mentioning that the total number of K-numbers and GO terms assigned to the rooibos protein dataset was 57,103 and 3.3 Mio, respectively; and that of those 4194 K-numbers 12,650 GO terms were unique.

With regard to K-number assignment, KAAS annotated only about ¼ of the sequences (15,616 transcripts) compared to EggNOG-Mapper (51,950 proteins). However, the number of unique K-numbers was 16% higher in the transcriptome dataset than in the protein dataset (Table 4). K-numbers permit classification of proteins into different protein families and link them to biological pathways, aka KEGG pathway modules. In this study, only plant-specific KEGG modules (258 modules found present in *Glycine max*, *Phaseolus vulgaris*, *Cicer arietinum*, *Oryza sativa*, *Theobroma cacao*, and/or *Arabidopsis thaliana*) were considered. The total numbers of plant-specific pathway modules identified by KAAS and EggNOG-Mapper were very similar: 208 and 199, respectively; and 60% of them were annotated to completion. Of the 11 parental pathway modules, all but “Xenobiotic degradation” had at least one complete submodule. In terms of complete submodule annotation, KAAS outperformed EggNOG-Mapper in all but two parental pathway modules: “Biosynthesis of terpenoids and polyketides”, and “Biosynthesis of other secondary metabolites”. Well-annotated pathways included carbohydrate, energy, nucleotide, lipid, cofactors and vitamin metabolism, as well as biosynthesis of terpenoids, polyketides and other secondary metabolites, where the overwhelming majority of the submodules were identified. In contrast, only 64% to 77% of the submodules for amino acid and glycan metabolism were represented in the datasets.

### 3.4. Orthology Analysis

**Figure 3:**
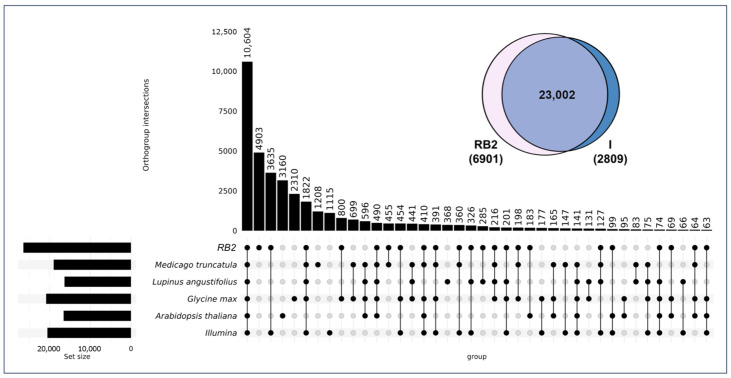
UpSet plot representing the number of orthogroups that are dataset-specific or shared between the RB2 and Illumina (I) rooibos datasets, and the protein datasets from four other plant species (black circles indicate presence of the dataset, the number above the column represents the number of OGs). Inset shows shared and dataset-specific orthogroups when the two rooibos datasets were investigated individually.

In a previous study, we reported on transcriptome assemblies from rooibos plants sequenced using Illumina [26]. To compare the ONT-based transcriptome generated here with the published Illumina transcriptome of the same growth type, the Illumina dataset A (hereafter referred to as I) was first reanalysed using TransDecoder, yielding 56,500 proteins. Subsequent analyses using OrthoFinder produced 32,712 OGs, clustering 87% of the RB2 and 84% of the Illumina protein datasets. In total, 70% of the OGs were shared between the two datasets, while 21% and 9% of them were specific to the RB2 and Illumina assemblies, respectively (Figure 3, inset).

When including other legume species and *A. thaliana* as an outgroup, a total of 365,820 proteins were assigned to 37,769 OGs. Of those, 10,604 OGs comprised sequences from all datasets (Figure 3). The RB2 proteome had the highest number of dataset-specific OGs (4903). Another 3635 OGs comprised only proteins from the two rooibos datasets (i.e., they are potentially rooibos-specific). Furthermore, 1822 OGs were shared among the legumes and 3160 OGs were specific to arabidopsis. Interestingly, a substantial number of proteins may be missing from the rooibos datasets: 596 OGs were found only in the four non-rooibos datasets, and an additional 441 OGs were shared between the other three legume species.

Including the additional plant species into the OG analyses improved grouping of the rooibos proteins (Figure 3; Table 5): the proportion of grouped proteins increased (by 7% in RB2 and 4% in I), the total number of OGs was reduced (by 11% in RB2 and 20% in I), and the number of dataset-specific OGs was also lowered (by 29% in RB2 and 38% in I). The formation of OGs where either RB2 or I proteins were grouped with proteins from at least one other plant species supports the notion of evolutional conservation and biological relevance of these rooibos sequences.

### 3.5. Differential Expression Analysis

For differential expression analysis, the processed reads of the L109, L111, R109, and R111 datasets were mapped separately to the functionally annotated RB2 transcriptome. The two leaf datasets and the two root datasets were used as technical repeats for the given plant tissue. With 99%, nearly all reads mapped back (Table 6). EdgeR and DESeq2 produced very similar results: of the 3248 transcripts predicted to be differentially expressed, 2677 (82%) were identified by both programs, of which 2453 transcripts were overexpressed in the leaves and 224 in the roots. EdgeR predicted fewer overexpressed transcripts in the more balanced leaf dataset than DESeq, and about the same number of overexpressed transcripts in the heavily unbalanced root dataset (Table 7).

While few of the overexpressed transcripts were annotated by KAAS (14% and 3% in the leaves and roots, respectively), most (71–83%) were annotated by EggNOG-Mapper (Table 7). The EggNOG-Mapper annotations for the shared dataset were therefore chosen as the basis for KEGG enrichment analyses.

### 3.6. Functional Enrichment Analyses

**Figure 4:**
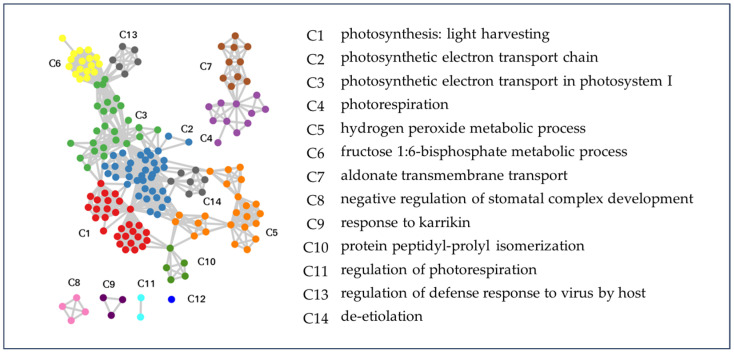
Network depicting the top 14 clusters of enriched GO terms in the leaves. Clusters are labelled based on the GO term with the lowest *p*-value, i.e., the term most significantly associated with the cluster. Each dot represents a GO term and the grey lines show the connections between the terms and clusters.

For the leaf and root datasets, enrichment analyses identified 340 and 83 enriched GO terms, associated with 1344 and 94 overexpressed transcripts, respectively. After clustering the redundant terms from the same biological processes, the leaf dataset produced a network of 29 GO term clusters (14 are shown in Figure 4), comprising 213 GO terms and 931 transcripts. Most GO terms were associated with photosynthesis (C1–C3; 473 transcripts), photorespiration (C4, C11; 94 transcripts), starch metabolism (C6; 52 transcripts), and plant growth regulation (e.g., C5, C8, C9; 114 transcripts).

**Figure 5:**
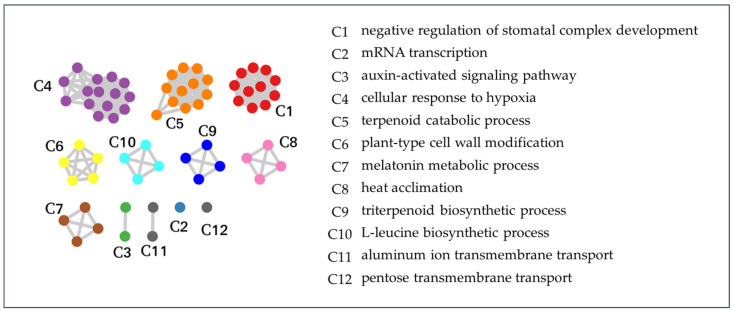
Network depicting the 12 clusters of enriched GO terms in the roots. GO term with the lowest *p*-value, i.e., the term most significantly associated with the cluster. Each dot represents a GO term and the grey lines show the connections between the terms and clusters.

The root dataset produced a network of 12 clusters comprising 65 GO terms (Figure 5). Of the 56 transcripts grouped in this analysis, eight were associated with regulation of stomata complex development (C1), seven with auxin-activated signalling (C3), five with melatonin metabolism (C7), eight with stress response (C4, C8), and eight with terpenoid metabolism (C5, C6).

**Figure 6:**
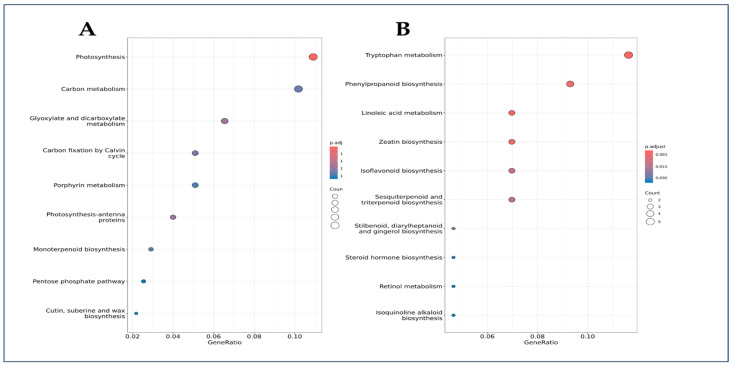
Dot-plots visualising the enriched KEGG pathways in (**A**) leaves and (**B**) roots. The dot sizes are proportional to the number of upregulated transcripts in the pathway, and the ’GeneRatio’ is the proportion of those transcripts.

Over-representation analysis of KEGG pathways found that 240 and 84 pathways were enriched in the leaves and roots, respectively (Figure 6). As expected, the most enriched pathways in the leaf dataset were ‘photosynthesis’ and ‘carbon metabolism’. The most enriched pathways in the root dataset were associated with the biosynthesis of plant hormones (tryptophan metabolism), stress response (linoleic acid and zeatin metabolism), and metabolism of secondary plant metabolites (phenylpropanoid and terpenoid biosynthesis).

In total, the overexpressed leaf and root datasets comprised 489 and 83 Pfam annotations, respectively. The top 10 most abundant terms are shown in Table 8. The leaf dataset was characterised by domains and families primarily associated with energy capture, photorespiration and carbon fixation (Chloroa_b-bind, RUBISCO, FBPase); and secondary metabolite biosynthesis (Thi4, UDPGT, Terpene_synth, Terpene_synth_C, p450); whereas the root dataset included domains and families potentially involved in microbe–host interaction and defence (Inhibitor_I9, PA, Peptidase_S8, Bet_v_1), root growth (BURP, DUF296), and secondary metabolite production (p450).

## 4. Discussion

### 4.1. MinION Sequencing and Data Processing

In this study, we successfully established Oxford Nanopore transcriptome sequencing for the recalcitrant plant species *A. linearis* using cDNA library construction kits. Three of the sequencing runs yielded between 12 Mio and 20 Mio basecalled raw reads at min QS = 7 (the lower number of reads generated for R109 was associated with the damage of 25% of the pores during flow cell priming and is therefore not a representative result). When using the SQK-PCS109 kit on MinION flow cells, previous studies reportedly generated between 3.5 Mio and 10.6 Mio reads [46,47,48]. The better results for L109 (12 Mio reads) reflect the good quality of the RNA and laboratorial procedures, but are likely also associated with improvements in the basecalling algorithms. The newer iteration of the cDNA sequencing kit (SQK-PCS111) was only used in combination with PromethION flow cells, which have six times more pores than the MinION flow cells (12,000 vs. 2048). Nonetheless, for MinION flow cells, the read numbers achieved with SQK-PCS111 (18 Mio) were very high indeed.

Extra care must be taken when filtering datasets for rRNA sequences, as SortMeRNA may remove valid mRNA sequences when employed in default mode. By using the plant-specific Magnoliophyta sequence database of large and small rRNA subunits from SILVA, an e-value threshold of 1 × 10^−10^, and validating the matches using Minimap2, average mRNA read recovery per dataset was increased from 74% (SortMeRNA default) to 80%, amounting to an additional 2.5 Mio reads.

### 4.2. Transcriptome Assembly

A number of tools have been developed for long-read transcriptome assembly. Most of these are algorithms that use a reference genome and corresponding gene annotations to guide transcript identification and assembly (e.g., StringTie2 [49], IsoQuant [50], Bambu [51], Mandalorion [52], FLAIR2 [53], LyRic [54], and IsoRefiner [55]). Ab initio assemblers that do not require a genome include RATTLE, RNA2-Bloom2, and isONform [56]. Rooibos is not a model organism. Although a first assembly of the rooibos genome has been generated [25], it is still very fragmented, error prone and redundant, and therefore not yet suitable to serve as a reference genome for transcriptome reconstruction. Initially, all three ab initio pipelines were investigated in this study; however, isONclust3 (the first step of the isON pipeline) consistently failed to complete the analyses, which was also reported in a previous study when investigating large, heterogenous datasets [57]. Therefore, in this study, only RATTLE and RNA-Bloom2 were compared.

The two assemblers differed substantially in their numbers of assembled transcripts. RATTLE generated on average 57,884 ± 614 transcripts, a number that is similar to the transcript counts reported for closely related and/or well-studied Fabaceae species (*Lupinus angustifolius* ≈ 58,000, *Medicago truncatula* ≈ 52,000, *Glycine max* ≈ 88,000; as per NCBI RefSeq genome annotations, accessed January 2026). However, assembly completeness was wanting, as indicated by a relatively low score for complete BUSCOs (only 79%). RNA-Bloom2 produced nearly four times as many transcripts (219,106 ± 7287 transcripts). The proportion of complete BUSCOs was substantially higher than in the RATTLE assembly (88–90%), but so was the proportion of duplicated BUSCOs (≈15% in RATTLE vs. ≈60% in RNA-Bloom2). The high number of transcripts and excess of duplicated BUSCOs implied high redundancy and the presence of artefacts in the RNA-Bloom2 assembly.

De Bruijn Graph assemblers (like RNA-Bloom2) often fail to collapse somewhat dissimilar sub-sequences, which can arise from reads rich in sequencing errors, but also from transcriptional noise (e.g., pre-mRNAs, unproductive transcripts that fail to produce functional proteins, and degraded mRNA molecules). RNA-Bloom2 has been shown to produce highly redundant transcriptome assemblies before [55,57]. Major sources of redundancy were found to be retention of truncated transcripts (i.e., incomplete assembly), and assembly of reads into a large number of possible isoforms, many of which may not actually exist (false positives). Redundancy was found to be more pronounced for highly expressed transcripts [58]. To reduce redundancy, clustering isoforms has have been suggested [57,59,60].

The most common tool used for post-assembly transcriptome clustering that generates consensus sequences is CD-HIT [61,62,63]. Because this algorithm is computationally expensive, we also tried VSEARCH. Overall, clustering of the RNA-Bloom2 assemblies reduced the number of transcripts by 20% (CD-HIT) and 26% (VSEARCH), respectively. After clustering, the average complete BUSCO scores were reduced by 0.9% (CD-HIT) and 2.3% (VSEARCH). Comparison of read length distributions before and after clustering revealed that both programs collapsed shorter transcripts (<1 kb) equally stringent, and that CD-HIT retained more of the longer transcript than VSEARCH. This may explain the higher complete BUSCO scores for the CD-HIT clustered datasets.

Even after clustering, the proportion of duplicated BUSCOs in the RNA-Bloom2 assemblies remained high (47% in the VSEARCH, and 53% in the CD-HIT datasets). In plants, on average 16.75% of the BUSCOs are duplicated [64]. Therefore, the clustered RNA-Bloom2 datasets must still be considered redundant and likely to include misassemblies. However, not all novel transcripts in these datasets are artefacts. Recent transcriptome studies that focused on the analysis of gene isoforms in plants indicate that transcript numbers are higher than estimated previously [65,66,67]. For the model organism arabidopsis, one of the latest published transcriptomes generated using PAC-BIO’s Iso-Seq platform comprised over 169,000 valid transcripts [68]. Of those, only half encoded proteins, another 40% were annotated as unproductive (i.e., transcripts that failed to produce functional proteins), and 9% of the transcripts were found to be non-coding. In this study, the datasets were not filtered for isoforms nor for unproductive/non-coding sequences, which may have also contributed to the high proportions of duplicated BUSCOs.

Considering the substantially higher proportion of complete BUSCOs, the polished, rRNA-depleted RNA-Bloom2 assembly clustered using CD-HIT (hereafter referred to as RB2) that comprised 169,122 transcripts and 95,054 proteins (80% of which were predicted to be full length) was used for downstream analyses.

### 4.3. Functional Annotations

Functional annotation showed that the RB2 assembly comprised informative sets of transcript and protein sequences. With 94%, nearly all proteins could be annotated using EggNOG-Mapper. KAAS and EggNOG-Mapper complemented each other with regard to pathway annotations, linking the transcripts and proteins to a total of 213 plant-specific pathways. The 129 pathways annotated to completion included essential housekeeping pathways associated with energy, lipid, nucleotide, amino acid, glucan, cofactor and vitamins, as well as terpenoid and polyketide metabolism. The 45 plant-specific pathways not represented in the rooibos transcriptome were found to be associated with energy (13), amino acid (14), glucan (10) and xenobiotic degradation (1) metabolism pathways in prokaryotes, indicating that the rooibos transcriptome generated in this study is likely free of bacterial transcripts.

### 4.4. Orthology Analyses

In a previous study, an Illumina transcriptome from leaves of a commercial rooibos plant had been assembled using IDBA-Trans, hereafter referred to as I [26]. With 91,171 transcripts (≥300 b, including rRNA), it yielded about half the number of transcripts obtained for the unclustered RB2 assemblies. Nonetheless, the proportion of complete BUSCOs (90%) was just as high. Re-analysis using the latest version of TransDecoder showed that here, too, only 62% of the transcripts encoded proteins (56,500), 71% of which were full length. Although the RB2 assemblies had longer transcripts, the protein sequences were longer in the Illumina dataset. While the first effect is likely a direct result of using long-read sequencing technologies and selecting for full-length transcripts, the longer protein lengths and higher proportions of truncated proteins in the Illumina dataset may reflect a short-read assembler-related bias towards maximising the assembled transcript length at the expense of shorter transcript versions that would encode full-length yet shorter proteins.

The majority of the rooibos proteins from RB2 (55%) and I (70%) were assigned to 23,002 OGs, shared between the two datasets. A surprisingly large number of the RB2 proteins (30,842; 32%) were clustered into 6901 RB2 dataset-specific OGs. Interestingly, this subset did not include any of the transcripts overexpressed in the root samples; and most these proteins were associated with a wide range of general housekeeping pathways. When adding other plant species to the orthology analysis, the numbers of RB2 dataset-specific OGs and their associated proteins were both reduced by ≈30%, confirming biological conservation of the now grouped rooibos sequences. Again, annotations of the protein sequences in the RB2 dataset-specific OGs did not indicate enrichment for any metabolic pathways or GO terms. However, both orthology analyses revealed that the proteins in the RB2-specific OGs were substantially shorter (RB2 vs. I: 200 amino acids; RB2 vs. ALL: 200 amino acids) than the BR2 proteins that grouped with sequences from other datasets (RB2 vs. I: 318 amino acids; RB2 vs. ALL: 294 amino acids). Considering that 80% of these RB2 protein sequences were predicted to be full length, future studies using, e.g., an improved version of the rooibos genome should investigate to what degree they represent artefacts or valid short proteins that may have been missed due to a general tendency to focus on the assembly of long transcripts.

### 4.5. Analysis of Differentially Expressed Transcripts and Pathway Enrichment Analyses

In this study, we investigated whether the read files generated from the four libraries can be used for comparative transcriptome analyses and permit identification of differentially expressed transcripts in the leaf and root datasets, respectively. For RNA-seq experiments, a minimum of three biological replicates has been recommended [69]. Since this study focussed primarily on sequencing method establishment, here only two technical replicates from the leaves and roots of a single seedling (i.e., no biological replicates) were analysed. Although the results obtained from our differential expression analyses can not be generalised due to the high likelihood of false positives, they do provide a first insight into the genes that may be differentially expressed in the leaves and roots of this particular rooibos seedling.

The two most common tools employed for differential gene expression analyses of long-read data are EdgeR and DESeq2 [60,70,71,72]. Both methods use a negative binomial distribution to model RNA-seq counts, but differ in their approaches to data normalisation: EdgeR employs trimmed mean of M-values, while DESeq2 uses median-based normalisation [73]. Of the two, EdgeR is considered more suitable for experiments with small sample sizes, i.e., low replicate counts [74]. In this study, the two programs shared a high number of transcripts predicted to be overexpressed in the leaves (2453; 83%) and roots (290; 77%), respectively. The large discrepancy in differentially expressed transcript numbers observed between the leaf and root datasets was likely associated with the substantially smaller number of reads produced from the R109 library, which resulted in an unbalanced root dataset and ultimately reduced the statistical power to detect overexpressed transcripts in these plant tissues. The shared overexpressed transcripts were subsequently used for functional enrichment analyses.

Considering that the study compared leaf and root samples, we expected to find transcripts associated with photosynthesis to be overexpressed in leaves, and transcripts associated with root growth and root-specific biological processes to be overexpressed in roots. The results of our differential expression analyses validated the chosen analysis approach, as many differentially expressed transcripts were predicted to be involved in biological processes specific to their respective tissues. Combined, the results from the over-representation analyses of GO terms, KEGG pathways and Pfam annotations facilitated identification of processes activated in the leaves and roots of the rooibos seedling.

The dataset of rooibos transcripts overexpressed in leaves was dominated by GO terms, KEGG pathways, and Pfam domains associated with photosynthesis, carbon fixation and photorespiration. The most abundant Pfam domain in the rooibos leaf dataset was the Chloroa_b-bind domain, a characteristic structural feature of the light-harvesting chlorophyll-binding proteins. Members of this protein family are among the most highly expressed genes in leaf tissues of plants, and have well-studied roles in light-harvesting complex assembly and energy transfer to photosynthetic reaction centres. Other domains over-represented in the overexpressed rooibos leaf dataset included RuBisCO and Fructose-1,6-bisphosphatase, both of which are known to be involved in carbon fixation, as well as the FMN_dh and Thi4 domains that are associated with photorespiration.

The dataset of rooibos transcripts overexpressed in roots was dominated by GO terms, KEGG pathways, and Pfam domains mainly associated with hormone production, stress regulation and defence. The most enriched KEGG pathway was tryptophan metabolism. Besides being involved in protein synthesis, tryptophan is arguably one of the most important precursors for the biosynthesis of very diverse primary and secondary metabolites. In roots, it is directly involved in the biosynthesis of auxin, the master regulator of root morphology [75], and melatonin, which promotes lateral root growth and root hair formation [76]. Concurrently, the GO terms “auxin-activated signalling pathways” and “melatonin metabolic process” were enriched in the root dataset. The most over-represented Pfam domain in the overexpressed rooibos root dataset was the Dimerisation, Methyltransf_2 domain, a structural feature of O-methyltransferases. Potentially, these rooibos enzymes may be involved in the biosynthesis of diverse secondary metabolites produced in roots, for example, in the enriched KEGG pathways “phenylpropanoid biosynthesis”, “isoflavonoid biosynthesis”, “sesquiterpenoid and triterpenoid biosynthesis”, “stilbenoid, diarylheptanoid and gingerol biosynthesis”, and “isoquinoline alkaloid biosynthesis”. So, O-methyltransferases have well-characterised roles in the biosynthesis of phenylpropanoids, flavonoids, alkaloids, and lignin [77], and members of this family have been reported to be overexpressed in root transcriptomes under normal conditions and in response to abiotic stresses [78,79].

### 4.6. Future Studies

Long-read RNA-seq analyses can be conducted using a range of different methods [74]. PacBIO is an alternative sequencing platform to ONT, which currently yields reads of higher quality. However, the ONT platform is substantially more user-friendly (cheaper, small devices, simple protocols). ONT itself offers different kits for transcriptome analyses, permitting direct RNA sequencing, direct cDNA sequencing and PCR-cDNA sequencing. Of these three technologies, the PCR-cDNA sequencing method currently yields the highest amount of reads and reportedly has a lower error rate compared to the other two ONT methods [57,80]. However, the ONT protocols are consistently being optimised and future studies should take alternative laboratorial approaches for RNA-seq analyses into account.

## Figures and Tables

**Table 1:** RNA extraction and read statistics for four sequencing libraries from leaves (L) and roots (R) of a rooibos seedling, sequenced using the PCS-SQK109 and PCS-SQK111 kits from ONT.

Raw	RNA (ng/µL)	Reads Sequenced	Basecalled Raw Reads (min QS 7)	Basecalled Raw Reads % (min QS 7)	Basecalled Data Size (Gb)	Mean Read Length	Median Read Length	Median Quality Score
L109	31.2	18,311,191	11,963,845	65.3	9.8	817.2	534	10.9
R109	41.2	7,719,243	5,995,604	77.7	5.1	843.1	512	11.3
L111	37.3	21,825,329	15,789,537	72.3	19.1	1207.4	1041	11.5
R111	35.7	25,583,014	19,832,193	77.5	17.6	887.1	749	11.7
**Q-Processing**	**# Reads After Pychopper**	**Median Read Length After Pychopper**	**# Reads After Cutadapt**	**Median Read Length After Cutadapt**	**# Reads After NanoFilt (>200 b)**	**Reads After NanoFilt (% of Basecalled)**	**Median Read Length After NanoFilt**	**Median Quality Score After NanoFilt**
L109	9,394,759	288	9,394,759	269	5,387,391	45.0	488	12.8
R109	4,511,855	201	4,511,855	182	2,190,249	36.5	400	13.4
L111	14,767,712	796	14,767,712	755	13,851,569	87.7	777	12.9
R111	18,774,932	529	18,774,932	493	16,199,757	81.7	550	13.6
**rRNA Filtering**	**# Reads After SmRNA (Default)**	**% Reads After SmRNA (Default)**	**# Reads After SmRNA (1 × 10^−^** **^10^** **,** **(Magnoliophyta)**	**% Reads After SmRNA (1 × 10^−^** **^10^** **, (Magnoliophyta)**	**# Reads After MM2 (Magnoliophyta)**	**% Reads After MM2 (Magnoliophyta)**	**# Reads After SmRNA & MM2 (Magnoliophyta)**	**% Reads After SmRNA & MM2 (Magnoliophyta)**
L109	4,284,929	79.5	4,557,585	84.6	4,586,847	85.1	4,509,777	83.7
R109	1,405,539	64.2	1,478,080	67.5	1,480,983	67.6	1,460,915	66.7
L111	11,170,863	80.6	12,779,170	92.3	12,842,968	92.7	12,354,839	89.2
R111	11,706,932	72.3	15,389,492	95.0	15,405,172	95.1	12,739,324	78.6

# = number; SmRNA = SortMeRNA; MM2 = Minimap2.

**Table 2:** Statistics of the 14 transcriptomes assembled using RATTLE and RNA-Bloom2 (RB2) for complete (rRNA+) and rRNA-filtered (rRNA−) datasets.

	Unclustered						VSEARCH				CD-HIT			
	rRNA+	rRNA−	rRNA+		rRNA−		rRNA+		rRNA−		rRNA+		rRNA−	
	RATTLE	RATTLE	RB2	RB2 p *	RB2	RB2 p *	RB2	RB2 p *	RB2	RB2 p *	RB2	RB2 p *	RB2	RB2 p *
**# Contigs**	57,270	58,497	226,681	226,073	212,443	211,225	168,100	165,233	158,887	156,126	181,973	179,573	171,419	169,122
**Mean read length**	1846	1842	1422	1418	1442	1439	1331	1321	1347	1339	1420	1414	1435	1429
**Median read length**	1640	1637	1245	1241	1266	1263	1133	1125	1146	1138	1231	1223	1245	1241
**Max read length**	10,356	9901	13,987	12,408	11,632	11,054	12,468	9708	11,244	9869	13,987	12,408	11,632	11,054
**BUSCOs:**														
**Complete**	78.5	78.7	87.9	90.1	87.7	89.9	85.4	87.8	85.6	87.7	86.8	89.3	86.8	89.2
**-Single-copy**	62.3	63.2	26.1	24.6	26.5	25.4	39.6	39.6	39.8	39.5	34.9	33.7	35.3	34.6
**-Duplicated**	16.2	15.5	61.8	65.5	61.2	64.5	45.8	48.2	45.8	48.2	51.9	55.6	51.5	54.6
**Fragmented**	5.6	5.5	2.3	1.3	2	1.4	2.8	2.1	2.6	1.9	2.7	1.6	2.4	1.6
**Missing**	15.9	15.8	9.8	8.5	10.2	8.7	11.8	10.1	11.9	10.4	10.5	9.1	10.8	9.1
**Computer resources:**														
**Wall time (HH:MM:SS)**	58:50:00	47:14:00	09:30:00	09:51:00	07:39:00	08:23:00	00:00:12	00:00:27	00:00:30	00:00:46	183:33:00	166:52:00	243:01:00	243:07:00
**Total CPU time**	1793 h	1658 h	338 h	365 h	256 h	299 h	24 s	11 s	52 s	28 s	183 h	167 h	239 h	239 h
**# CPU cores**	56	56	56	56	56	56	1	1	1	1	1	1	1	1

# = number; * = polished using Illumina data.

**Table 3:** Summary of the RB2 rRNA− polished CD-HIT (RB2) assembly annotations.

	RB2
**Number of transcripts**	169,122
**Number of transcripts annotated using KAAS**	15,616
**Number of candidate proteins**	95,054
**Number of proteins annotated using Swissprot (blastp)**	87,340
**Number of proteins annotated with EggNOG:**	89,492
- **Annotated with at least one K-number**	51,950
- **Annotated with at least one GO term**	54,885
- **PFAM-A annotations**	82,794

**Table 4:** KAAS vs. eggNOG KEGG annotation summary.

	KAAS (Transcript Dataset)	EggNOG-Mapper (Protein Dataset)
**Sequences with at least one K-number**	15,616	51,950
**Total K-numbers**	15,616	57,103
**Unique K-numbers**	4872	4194
**Total KEGG modules (plant-specific)**	208	199
**Complete KEGG modules (plant-specific)**	125	119
**Incomplete KEGG modules (plant-specific)**	83	80
**Parental pathway modules and total number of submodules (plant-specific)**	**Annotated submodules compl./incompl./total**	**Annotated submodules compl./incompl./total**
**Carbohydrate metabolism (30)**	17/11/28	17/9/26
**Energy metabolism (32)**	14/2/16	14/3/17
**Lipid metabolism (20)**	14/4/18	12/6/18
**Nucleotide metabolism (11)**	7/4/11	7/4/11
**Amino acid metabolism (59)**	27/18/45	26/17/43
**Glycan metabolism (44)**	15/19/34	13/15/28
**Metabolism of cofactors and vitamins (36)**	17/18/35	13/21/34
**Biosynthesis of terpenoids and polyketides (13)**	10/2/12	12/0/12
**Biosynthesis of other secondary metabolites (8)**	2/4/6	4/4/8
**Xenobiotics degradation (1)**	0/0/0	0/0/0
**Signature modules (4)**	2/1/3	1/1/2

**Table 5:** Number of OGs shared between the proteins of the RB2 (RB2 rRNA− polished CD-HI) and the I (Illumina) rooibos transcriptomes, and the protein datasets of other plant species (*L. angustifolius*, *M. truncaluta*, *G. max*, and *A. thaliana*).

	Proteins	Proteins in OGs (%)	RB2	I	LA	MT	GM	AT
**RB2**	95,054	95	26,496	18,684	14,354	15,297	15,926	12,228
**I**	56,500	88	18,684	20,585	13,560	14,286	14,522	11,601
**LA**	33,083	94	14,354	13,560	16,363	14,735	14,908	12,096
**MT**	44,450	86	15,297	14,286	14,735	19,035	16,393	12,699
**GM**	88,412	90	15,926	14,522	14,908	16,393	20,896	12,855
**AT**	48,321	93	12,228	11,601	12,096	12,699	12,855	16,593

**Table 6:** Mapping statistics for the four read datasets when aligned to the transcriptome.

	# of Transcripts Hit	# of Reads Mapped	% Reads Mapped
**L109**	135,305	3,684,428	99
**R109**	115,853	1,144,162	99
**L111**	143,272	11,864,639	99
**R111**	142,272	11,302,076	99

**Table 7:** Annotation summaries for the transcripts upregulated in the leaves and the root samples.

	EgdeR		DESeq2		Shared	
	Leaves	Roots	Leaves	Roots	Leaves	Roots
**Significantly overexpressed**	2537	261	2875	253	2453	224
**Annotated by KAAS:**	340	8	408	7	330	7
**Annotated by EggNOG-Mapper:**	2096	186	2360	180	2034	161
**-With K-numbers**	1278	70	1448	67	1243	62
**-With GO terms**	1388	106	1586	104	1344	94

**Table 8:** Top 10 most abundant Pfam terms in the overexpressed leaf and root datasets.

Pfam Domains in Leaf Dataset	Protein Counts	Pfam Domains in Root Dataset	Protein Counts
Chloroa_b-bind	125	Dimerisation, Methyltransf_2	12
RbcS, RuBisCO_small	34	DUF640	8
FBPase	30	Inhibitor_I9, PA, Peptidase_S8	8
Lipase_GDSL	29	p450	8
FMN_dh	28	EamA	7
Thi4	26	Bet_v_1	6
Terpene_synth, Terpene_synth_C	24	BURP	6
UDPGT	24	peroxidase	5
Pro_CA	23	DUF296	4
p450	21	Tryp_alpha_amyl	4

## Data Availability

The data presented in this study are available on request from the corresponding author. The datasets generated and analysed in this article cannot be submitted to public databases due to the restrictive Biodiversity Legislation of South Africa.
